# Zygomaticomaxillary modifications in the horizontal plane induced by micro-implant-supported skeletal expander, analyzed with CBCT images

**DOI:** 10.1186/s40510-018-0240-2

**Published:** 2018-10-22

**Authors:** Daniele Cantarella, Ramon Dominguez-Mompell, Christoph Moschik, Luca Sfogliano, Islam Elkenawy, Hsin Chuan Pan, Sanjay M. Mallya, Won Moon

**Affiliations:** 1 Division of Oral Biology and Medicine, School of Dentistry, Center for Health Science, University of California, 10833 Le Conte Avenue, Box 951668, CA, Los Angeles 90095-1668 USA; 2 Division of Growth and Development, Section of Orthodontics, School of Dentistry, Center for Health Science, University of California, 10833 Le Conte Avenue, Box 951668, CA, Los Angeles 90095-1668 USA; 3 Division of Diagnostic and Surgical Sciences, Section of Oral & Maxillofacial Radiology, School of Dentistry, Center for Health Science, University of California, Room 53-068 B CHS, 10833 Le Conte Avenue, Box 951668, CA, Los Angeles 90095-1668 USA; 4 Division of Growth and Development, Section of Orthodontics, School of Dentistry, Center for Health Science, University of California, Room 63-082 CHS, 10833 Le Conte Avenue, Box 951668, CA, Los Angeles 90095-1668 USA

**Keywords:** Cone-beam computed tomography (CBCT), Zygomatic arch, Miniscrew-assisted rapid palatal expansion (MARPE), Maxillary skeletal expander (MSE), Bone-anchored maxillary expander (BAME), Miniscrew

## Abstract

**Background:**

Miniscrew-assisted rapid palatal expansion (MARPE) has been adopted in recent years to expand the maxilla in late adolescence and adult patients. Maxillary Skeletal Expander (MSE) is a device that exploits the principles of skeletal anchorage to transmit the expansion force directly to the maxillary bony structures and is characterized by the miniscrews’ engagement of the palatal and nasal cortical bone layers. In the literature, it has been reported that the zygomatic buttress is a major constraint that hampers the lateral movement of maxilla, since maxilla is located medially to the zygomatic arches. The objective of the present study is to analyze the changes in the zygomatic bone, maxillary bone, and zygomatic arches and to localize the center of rotation for the zygomaticomaxillary complex in the horizontal plane after treatment with MSE, using high-resolution cone-beam computed tomography (CBCT) images.

**Methods:**

Fifteen subjects with a mean age of 17.2 (± 4.2) years were treated with MSE. CBCT records were taken before and after miniscrew-assisted maxillary expansion; three linear and four angular parameters were identified in the axial zygomatic section (AZS) and were compared from pre-treatment to post-treatment using the Wilcoxon signed rank test.

**Results:**

Anterior inter-maxillary distance increased by 2.8 mm, posterior inter-zygomatic distance by 2.4 mm, angle of the zygomatic process of the temporal bone by 1.7° and 2.1° (right and left side) (*P* < 0.01). Changes in posterior inter-temporal distance and zygomaticotemporal angle were negligible (*P* > 0.05).

**Conclusions:**

In the horizontal plane, the maxillary and zygomatic bones and the whole zygomatic arch were significantly displaced in a lateral direction after treatment with MSE. The center of rotation for the zygomaticomaxillary complex was located near the proximal portion of the zygomatic process of the temporal bone, more posteriorly and more laterally than what has been reported in the literature for tooth-borne expanders. Bone bending takes place in the zygomatic process of the temporal bone during miniscrew-supported maxillary expansion.

## Background

The effects of rapid maxillary expansion (RME) on the midface have been studied throughout orthodontic history and were traditionally conducted on two-dimensional X-rays, like the lateral and posteroanterior cephalograms, or on dental casts [1–4]. Wertz studied maxillary expansion also with the aid of dried skulls and found that the maxillary halves inclined laterally during the expansion procedure, concluding that the maxillary rotational fulcrum in the coronal plane must be close to the frontomaxillary suture [2]. Additionally, he reported that the midpalatal suture opened in a non-parallel fashion, with the widest opening at the anterior nasal spine (ANS) and a decreasing split in the posterior palatal region, thus locating the maxillary rotational fulcrum in the horizontal plane close to the pterygopalatine suture. These findings were confirmed by the following studies [5–7]. Due to the nature of the methods utilized, only limited insight into the in vivo RME skeletal and dental effects were possible until the advent of the cone-beam computerized tomography (CBCT) in the dental field. With the CBCT, in fact, it became feasible to investigate the expansion effects in three dimensions, and as the resolution of the CBCT machines improved, not only the movement of maxillofacial bones became measurable, but also the effects on the maxillary and circum-maxillary sutures [5–10].

Miniscrew-assisted rapid palatal expansion (MARPE) devices have been developed with the purpose to increase orthopedic changes in the midface in orthodontic practice, especially in post-pubertal patients, and to reduce the negative repercussions on the periodontium of posterior teeth [11–17]. One such MARPE appliance, the Maxillary Skeletal Expander (MSE), features four miniscrews positioned in the posterior part of the palate which engage both the palatal and nasal cortical bone layers [11, 14, 18].

The aim of the present study was to analyze the zygomaticomaxillary modifications induced by the miniscrew-supported MSE and to localize the rotational fulcrum for the zygomaticomaxillary complex in the horizontal plane.

## Methods

### Study design

The study is retrospective and was approved by the Institutional Review Board (IRB).

### Participants and intervention

The sample comprised 15 patients (9 females, 6 males), with a mean age of 17.2 ± 4.2 years (range 13.9–26.2 years), all treated by means of MSE (Biomaterials Korea Inc.). Nine patients displayed bilateral posterior crossbite, five unilateral crossbite, and one maxillary transverse deficit but no dental crossbite. All treatments were conducted at the Orthodontic Clinic, and any bracket bonding or further appliance placement was performed only after completion of maxillary expansion using MSE.

### Inclusion criteria

The inclusion criteria were as follows: (1) transverse maxillary deficiency, diagnosed according to a modified version of Andrews’ analysis of six elements [19], as described below; (2) treatment plan comprising MSE; (3) CBCT scans taken, respectively: before treatment and within 3 weeks of active expansion completion; (4) no craniofacial abnormalities; and (5) no previous orthodontic treatment [14].

**Fig. 1:**
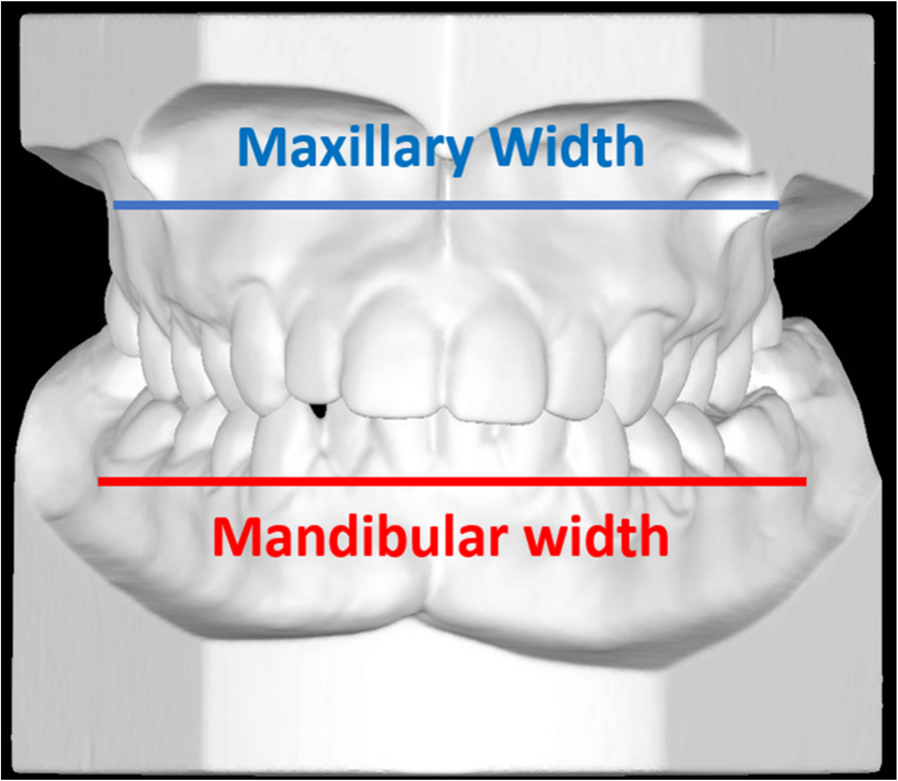
Maxillary and mandibular width, utilized to calculate the transverse maxillary skeletal deficiency

The relationship between the maxillary and mandibular widths was analyzed (Fig. 1). The maxillary width was taken as the distance between the most depressed points of maxillary vestibule at the level of the mesio-buccal cusp of first molars, whereas the mandibular width was the distance between the right and left WALA ridges at the mesio-buccal groove of the first molars. Maxillary skeletal transverse deficit was calculated as the difference between the mandibular and maxillary widths [14].

MSE, rather than a conventional tooth-borne palatal expander, was selected based on the following criteria: patient maturity (appearance of secondary sexual characteristics including facial hair, voice changes, menstrual cycle onset, and cervical vertebral maturity above stage CS4) [20], dolicofacial vertical pattern (high SN-GoGn and FMA angles), and history of nasal airway problems [14]. Indeed, the Section of Orthodontics preferentially treats dolicofacial patients with MSE, as bone-borne expanders generally result in less posterior mandibular rotation [21].

### Expander design and activation protocol

**Fig. 2:**
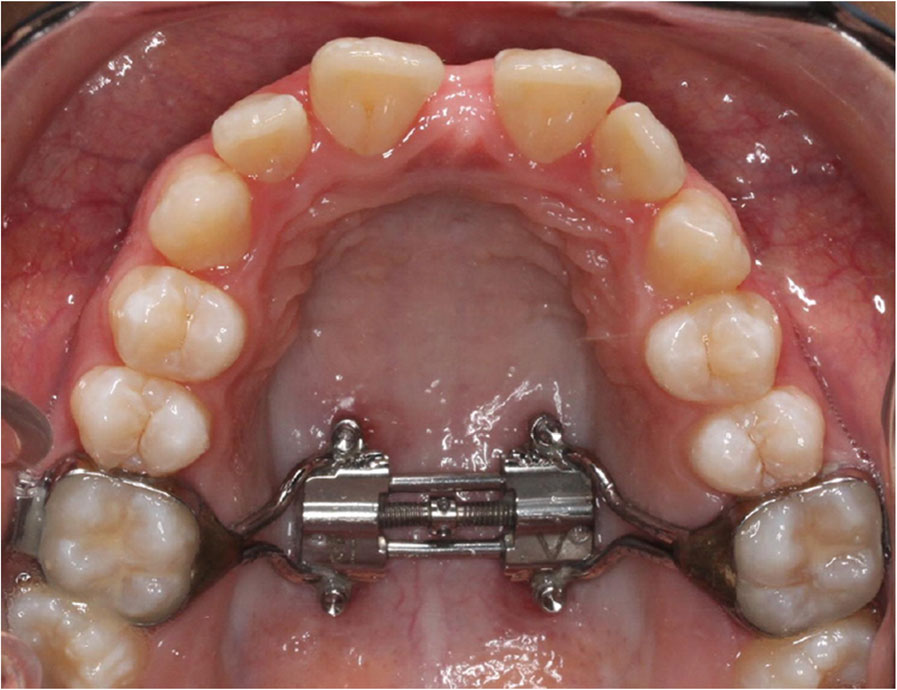
Intraoral picture of Maxillary Skeletal Expander (MSE)

The MSE device (Fig. 2) comprises an expansion jackscrew, whose body presents four slots for palatal miniscrews, and bilateral arms connected to molar bands [11, 14, 15]. For each patient, the length of miniscrews was chosen by measuring the bone thickness in the paramedian area of the palate at the level of maxillary first molars on pre-expansion CBCT, to ensure the miniscrews engagement of cortical bone layers of palatal vault and nasal floor. The diameter of miniscrews was 1.5 mm in all treated patients.

The rate of expansion was two turns per day (0.25 mm per turn) until a diastema appeared and then one turn per day. Expansion was completed when the maxillary skeletal width was equal to or greater than the mandibular width [14]. In order to retain the expansion achieved, MSE was kept in place without further activation for ≥ 3 months.

### 3D analysis

CBCT scans (NewTom 5G, with 18 × 16 field of view, 14-bit gray scale and standard voxel size 0.3 mm) were taken both before expansion and within 3 weeks of its completion, with a mean of 5 ± 2 months between the two radiologic exams (this time period included the time taken for appliance manufacture and delivery and administrative procedures) [14]. CBCT settings were 18-s scan time (3.6 s emission time) at 110 kV. The automated exposure control system enabled detection of the patient’s anatomical density, and the milliampere was adjusted accordingly.

**Fig. 3:**
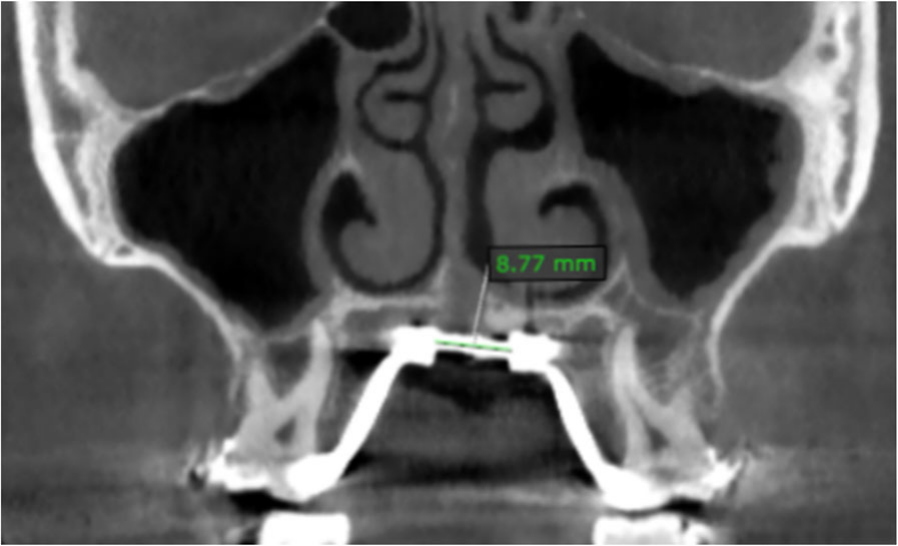
CBCT coronal section, showing the distance between the two halves of the MSE expansion jackscrew after expansion

The total MSE jackscrew activation for each patient was calculated as the distance between the two halves of the expansion screw measured on post-expansion CBCT (Fig. 3); the pre-expansion distance was determined by taking a CBCT scan of an MSE appliance and measuring the distance 10 times; the pre-expansion distance was subtracted from the post-expansion one, and values were then averaged to obtain the mean and standard deviation [14].

**Fig. 4:**
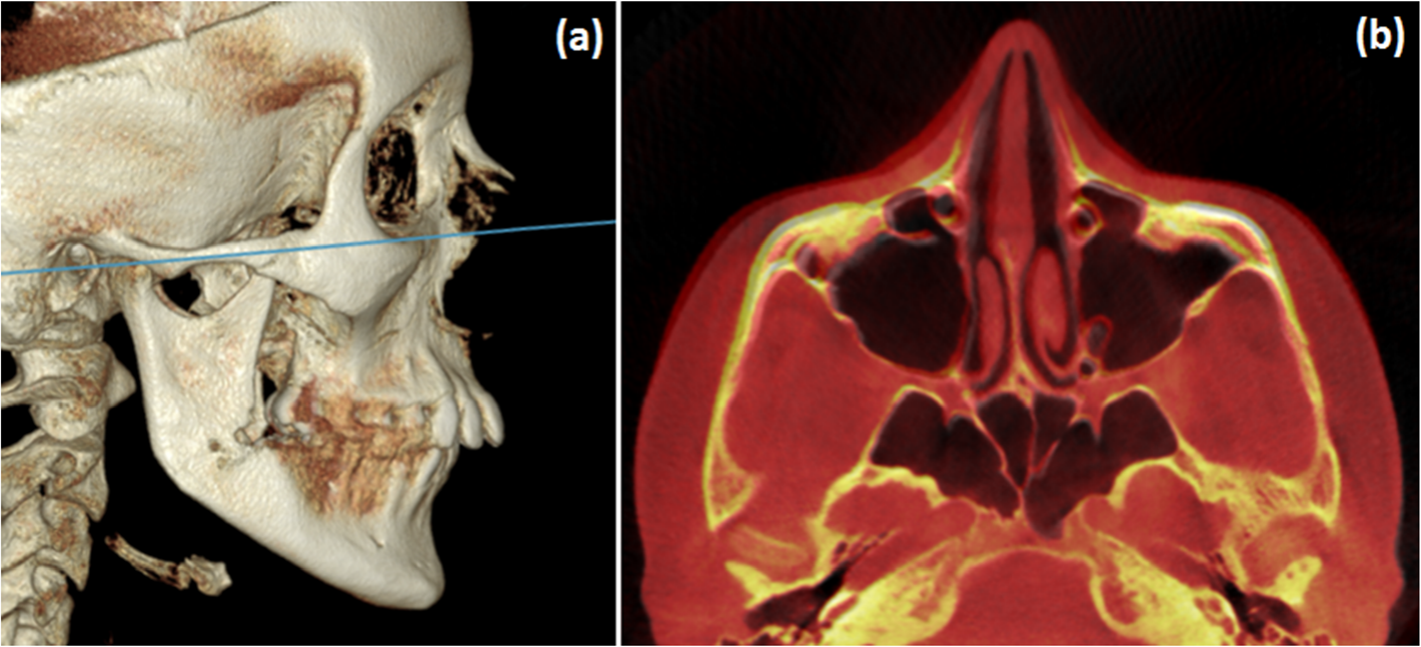
Axial zygomatic section (AZS). **a** Lateral view of 3D rendering, showing the AZS in blue. **b** Pre- and post-treatment superimposed image of a MSE patient

To analyze skeletal changes induced solely by MSE, post-expansion scans were taken before any bracket bonding or fitting of other appliances. Each post-expansion scan was superimposed on its corresponding pre-expansion scan on the stable structures of the anterior cranial base using OnDemand3D software and automated processing and matching of the voxel grey scale patterns [22–24]. The axial zygomatic section (AZS), passing through the vertical midpoint of the zygomaticotemporal sutures and the vertical midpoint of the articular tubercle of the temporal bones (TBATs) (Fig. 4), was used as a reference for three linear and four angular parameters for comparison in the pre- and post-expansion scans (Table 1).

**Table 1:** Parameters evaluated in the study

Linear measurements	
1	Anterior inter-maxillary distance (AIMD)
2	Posterior inter-zygomatic distance (PIZD)
3	Posterior inter-temporal distance (PITD)
Angular measurements	
4	Right zygomaticotemporal angle (Rt ZTA)
5	Left zygomaticotemporal angle (Lt ZTA)
6	Right angle of the zygomatic process of the temporal bone (Rt ZPA)
7	Left angle of the zygomatic process of the temporal bone (Lt ZPA)

*Rt* right, *Lt* left

**Fig. 5:**
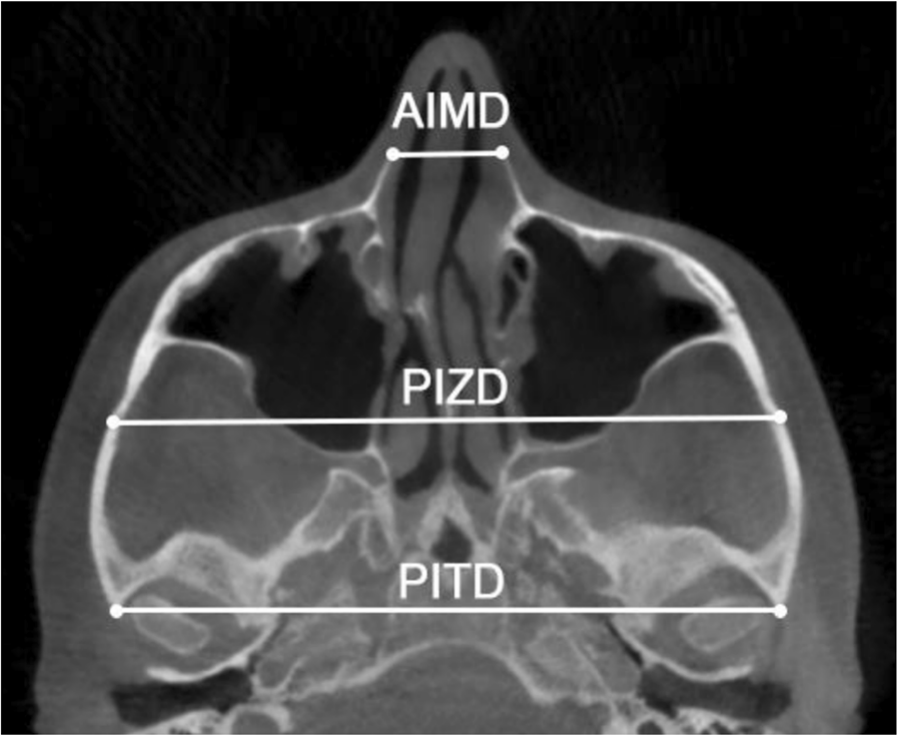
Skeletal linear measurements in the axial zygomatic section (AZS): anterior inter-maxillary distance (AIMD), posterior inter-zygomatic distance (PIZD), posterior inter-temporal distance (PITD)

**Fig. 6:**
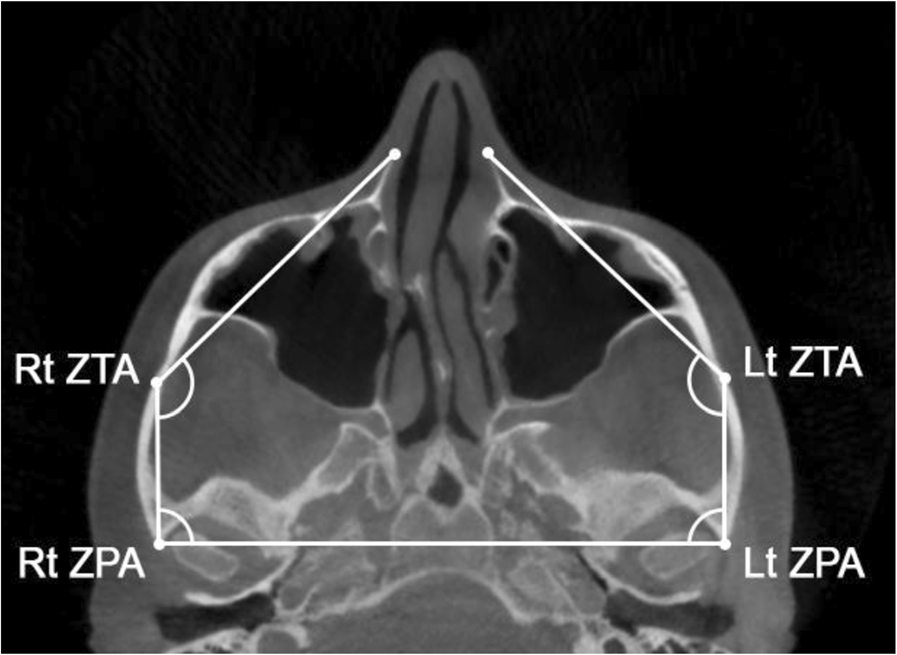
Skeletal angular measurements in the axial zygomatic section (AZS): zygomaticotemporal angle (ZTA), angle of the zygomatic process of the temporal bone (ZPA). Rt: right; Lt: left

Linear measurements (Fig. 5) included the anterior inter-maxillary distance (AIMD), from the most anterior point on the right maxilla to the most anterior point on the left maxilla; the posterior inter-zygomatic distance (PIZD), between the outermost points on the right and left zygomaticotemporal sutures, respectively; and the posterior inter-temporal distance (PITD), between the most posterior point on the left and right TBATs, respectively. Angular measurements (Fig. 6) were the zygomaticotemporal angle (ZTA), formed by the most anterior point on the maxilla, the most external point on the zygomaticotemporal suture, and the most posterior point on the TBAT; and the angle of the zygomatic process of the temporal bone (ZPA), formed by a line connecting the most posterior point of the left and right TBATs, and a line connecting the most posterior point on the TBAT to the most external point on the zygomaticotemporal suture. The ZTA and ZPA were used to analyze the rotation of the zygomaticomaxillary complex in the horizontal plane.

### Statistical analysis

Method reliability was assessed by obtaining measurements for all seven variables on eight randomly selected patients by two raters. Measurements were repeated after 2 weeks by the same operators after re-orientation of the skull on the reference plane (AZS). Indeed, reliability parameters are the combination of errors in reference plane identification and landmark location. Rater standard deviation and coefficient of variance; error standard deviation and coefficient of variation; and intra-class correlation coefficient (ICC) were calculated.

For each variable, the pre-expansion value was subtracted from the post-expansion value, and the mean change was compared to zero. *P* values were calculated using the Wilcoxon signed rank test for paired data. For all considered parameters, the confidence interval of treatment change (confidence level of 95%) has been calculated.

## Results

For the considered parameters, the rater coefficient of variation was 1.22 or less, and the error coefficient of variation was 1.97% or less (Table 2), showing that the reliability of the measurement method was very high.

**Table 2:** Analysis of method reliability

Parameter		Unit	Rater SD	Error SD	Rater CV (%)	Error CV (%)	ICC (%)
Linear measurements							
1	Anterior inter-maxillary distance (AIMD)	mm	0.24	0.39	1.22	1.97	96.7
2	Posterior inter-zygomatic distance (PIZD)	mm	0.35	0.72	0.31	0.64	95.9
3	Posterior inter-temporal distance (PITD)	mm	0.17	0.57	0.15	0.49	92.6
Angular measurements							
4	Right zygomaticotemporal angle (Rt ZTA)	°	0.82	1.14	0.61	0.85	93.9
5	Left zygomaticotemporal angle (Lt ZTA)	°	0.83	1.64	0.62	1.22	90.4
6	Right angle of the zygomatic process (Rt ZPA)	°	0.19	0.93	0.21	1.04	97.7
7	Left angle of the zygomatic process (Lt ZPA)	°	0.43	1.19	0.49	1.33	96.6

*SD* Dahlberg standard deviation, *Rater CV* rater coefficient of variation = rater SD/overall mean, *Error CV* error coefficient of variation = error SD/overall mean, *ICC* intra-class correlation coefficient = patient variance/total variance

The average amount of MSE jackscrew activation was 6.8 ± 1.9 mm, with a range of 4.1 to 10.5 mm. The period of active maxillary expansion ranged from 12 to 36 days.

With regard to the linear measurements (Table 3), the largest change was at the anterior inter-maxillary distance (AIMD), followed by the increase in the posterior inter-zygomatic distance (PIZD) (*P* < 0.01), while the modification in the posterior inter-temporal distance (PITD) was negligible and not statistically significant (P>0.05).

**Table 3:** Results for linear and angular measurements

		Unit	Before expansion		After expansion		Treatment change		95% CI for treatment change		
			mean	sd	mean	sd	mean	sd	Lower limit	Upper limit	*p* value
Linear measurements											
1	Anterior inter-maxillary distance (AIMD)	mm	17.05	3.06	19.81	3.11	2.76	1.51	1.92	3.60	< .0001**
2	Posterior inter-zygomatic distance (PIZD)	mm	111.80	4.99	114.20	5.34	2.40	0.58	2.08	2.72	< .0001**
3	Posterior inter-temporal distance (PITD)	mm	115.38	5.35	115.40	5.38	0.02	0.08	− 0.02	0.06	0.175
Angular measurements											
4	Right zygomaticotemporal angle (Rt ZTA)	°	134.20	5.81	134.10	6.08	− 0.10	1.09	− 0.70	0.50	0.612
5	Left zygomaticotemporal angle (Lt ZTA)	°	134.30	6.05	134.30	5.63	− 0.04	1.53	− 0.89	0.81	0.882
6	Right angle of the zygomatic process (Rt ZPA)	°	87.16	4.71	88.90	5.18	1.74	1.07	1.15	2.33	< .0001**
7	Left angle of the zygomatic process (Lt ZPA)	°	86.62	5.29	88.75	6.00	2.13	1.57	1.26	3.00	0.000**

*CI* confidence interval

**p < 0.01

In relation to the angular measurements (Table 3), the angle of the zygomatic process of the temporal bone (ZPA) significantly increased with MSE treatment (*P* < 0.01), while the zygomaticotemporal angle (ZTA) underwent negligible changes without statistical significance (*P* > 0.05).

For each parameter, the upper and lower limit of the confidence interval of treatment change (confidence level of 95%) is given in Table 3.

## Discussion

Several studies have reported that the opening of the midpalatal suture with tooth-borne palatal expanders is V-shaped with a larger split anteriorly and a progressively smaller split towards the posterior palatal region [2, 5–7]. Gautam et al. [25] reported in a finite element method (FEM) investigation with conventional rapid palatal expansion (RPE) that the maxillary center of rotation in the horizontal plane is located between the lateral and medial pterygoid plates. The pterygopalatine suture, due to the rigid interlock between articulating bones, cannot be split by tooth-borne expanders [9], and therefore, it acts like a hinge around which the maxillary halves rotate during the expansion, producing the V-shaped movement of maxilla.

In the present study, the anterior inter-maxillary distance (AIMD) increased by 2.7 mm and the posterior inter-zygomatic distance (PIZD) by 2.4 mm. These results show that the maxilla, the zygomatic bone and the whole zygomatic arch were significantly displaced in a lateral direction, after treatment with MSE.

The zygomatic process angle of the temporal bone (ZPA) increased by 1.7° and 2.1° on the right and left side respectively (*P* < 0.01). The zygomaticotemporal angle (ZTA) is a variable that indicates the relative inclination between the zygomaticomaxillary complex and the zygomatic process of the temporal bone. Changes at ZTA were negligible and without statistical significance, showing that the zygomaticomaxillary complex and the zygomatic process of the temporal bone maintained their relative inclination during maxillary expansion and they both rotate together around a common center of rotation.

**Fig. 7:**
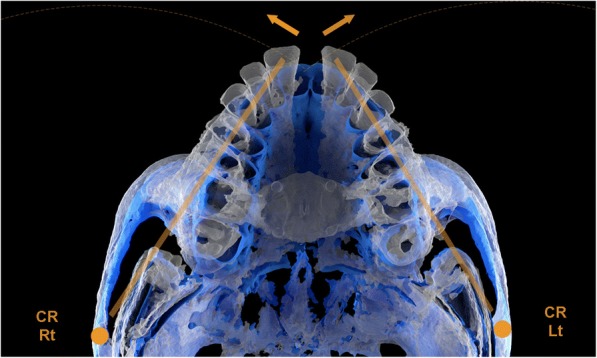
Superimposed 3D images of a MSE patient showing the rotation of the zygomaticomaxillary complex with a center of rotation (CR) located near the proximal aspect of the zygomatic process of the temporal bone. Blue: pre-expansion. White: post-expansion

Since the increase in the posterior inter-temporal distance (PITD) was negligible, and the increase in the posterior inter-zygomatic distance (PIZD) and in the zygomatic process angle (ZPA) of the temporal bone were of considerable magnitude, we conclude that the zygomaticomaxillary complex rotates around a center of rotation located in the proximal portion of the zygomatic process of the temporal bone (Fig. 7).

**Fig. 8:**
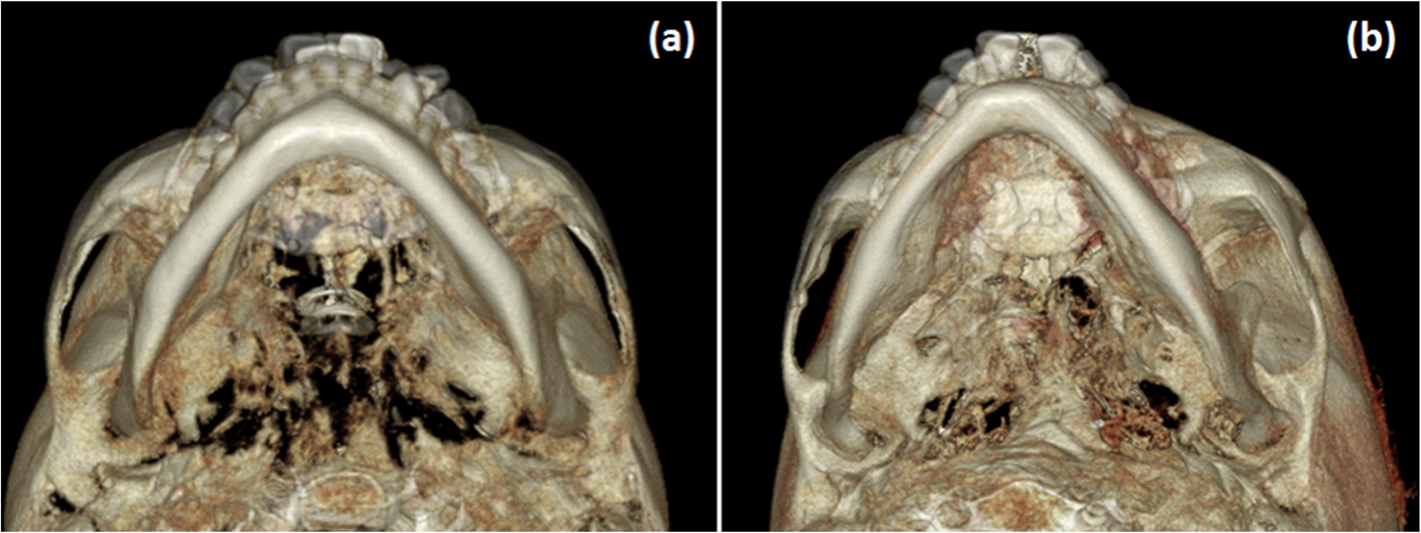
Superimposed 3D renderings of MSE patients showing the skeletal changes in the zygomaticomaxillary complex and zygomatic arch in a horizontal plane. **a** Lower view. **b** Lower 3/4 view

MSE, in contrast with tooth-borne expanders, has shown to be able to disarticulate the pterygopalatine suture and to produce an almost perfectly parallel opening of the midpalatal suture [14], indicating that the fulcrum for the maxillary rotation is located more posteriorly and more laterally than what has been reported for tooth-borne expanders, which is compatible with a location near the proximal portion of the zygomatic process of the temporal bone. This location of the maxillary rotational fulcrum can also explain the forward movement of the maxilla, frequently found in MSE patients (Figs. 7 and 8). The maxilla is located medially and anteriorly relative to this fulcrum. As the zygomaticomaxillary complex rotates outwards around the proximal portion of the zygomatic process of the temporal bone, the maxillary halves will initially move laterally and anteriorly (Fig. 7). This forward maxillary movement can also help in disarticulating the pterygopalatine suture during the maxillary expansion, as found in a previous study [14].

The significant displacement of the zygomatic arch is probably due to the mechanism of action of MSE. The appliance is positioned in the posterior part of the palate, to produce an expansion force vector in line with the zygomatic buttress bone [14] and utilizes four miniscrews with bicortical engagement to enhance the transmission of the device expansion force to the underlying bony structures [18]. This is in agreement with the finding of a midpalatal suture split in all treated patients in the present study (average suture opening was 4.8 mm at anterior nasal spine and 4.3 mm at posterior nasal spine), and with a negligible buccal tipping of maxillary first molars reported in a previous investigation [26].

The rotational fulcrum positioned at the proximal portion of the zygomatic process of the temporal bone can be explained by a bone-bending effect in this area. Bone bending is a phenomenon that takes place when a cyclical bending force is applied to a bone and is considered an adaptive mechanism to dissipate the energy in order to prevent an overt fracture [27]. Lateral loads applied to a bone produce tensile forces at the bone surface facing the load and compressive forces at the opposite surface, generating microfractures in the trabeculae of the cancellous bone [27, 28]. Microfractures subsequently activate self-repair mechanisms [29], leading to bone callus formation on the damaged trabeculae. Microfractures and self-repair through new bone formation progressively lead to a change in bone shape [27].

It has been reported that the bone resistance to a bending force depends on the density, calcium content, cortical to cancellous bone ratio, micro-architecture, and geometry of the bone [30, 31]. Regarding this last point, the resistance to bending is directly related to the third power of the bone diameter [32], and this can explain why the proximal portion of the zygomatic process of the temporal bone, that is one of the thinnest parts of the zygomatic arch, tends to bend during maxillary expansion and becomes the rotational fulcrum for the zygomaticomaxillary complex in the horizontal plane.

Further studies are needed to investigate how the diverse morphology of the zygomatic arch in different patients may affect the success rate of midface expansion, especially in adult patients, where bones may have a lower elasticity. In the present study, patients were at post-pubertal maturation stage, and age range (13.9–26.2 years) included late adolescence and young adulthood. In a previous investigation [14], it was found that for this age group, the magnitude of lateral maxillary movement, measured by the extent of midpalatal suture opening at anterior nasal spine and posterior nasal spine, had no correlation with age. One possible explanation can be that a reduced midface bone elasticity, especially in the zygomatic arch, may affect the lateral movement of maxilla in ages above 26 years, and this aspect needs further investigations.

Furthermore, differences in geometry of zygomatic arches between right and left side of the skull may play a certain role in explaining the asymmetry of maxillary movement reported in the literature [14], possibly along with other contributing parameters such as uneven bone density and suture interdigitation, nasal septum deviation, asymmetry in occlusal forces, and others.

## Conclusions

1. In the horizontal plane, the maxillary and zygomatic bones and the whole zygomatic arch were significantly displaced in a lateral direction after expansion using MSE
2. The center of rotation for the zygomaticomaxillary complex was located near the proximal portion of the zygomatic process of the temporal bone, more posteriorly and more laterally than what has been described in the literature for tooth-borne expanders
3. A significant bone bending takes place in the zygomatic process of the temporal bone during the miniscrew-supported maxillary expansion

## Abbreviations

AIMD: Anterior inter-maxillary distance
ANS: Anterior nasal spine
AZS: Axial zygomatic section
CBCT: Cone-beam computed tomography
CV: Coefficient of variation
ICC: Intra-class correlation coefficient
IRB: Institutional Review Board
MARPE: Miniscrew-assisted rapid palatal expansion
MSE: Maxillary Skeletal Expander
PITD: Posterior inter-temporal distance
PIZD: Posterior inter-zygomatic distance
RME: Rapid maxillary expansion
RPE: Rapid palatal expansion
SD: Dahlberg Standard Deviation
TBAT: Temporal bone articular tubercle
ZPA: Angle of the zygomatic process of the temporal bone
ZTA: Zygomaticotemporal angle
